# A Near-Infrared Light-Responsive Hybrid Hydrogel Based on UCST Triblock Copolymer and Gold Nanorods

**DOI:** 10.3390/polym9060238

**Published:** 2017-06-20

**Authors:** Hu Zhang, Shengwei Guo, Shangyi Fu, Yue Zhao

**Affiliations:** 1 Département de Chimie, Université de Sherbrooke, Sherbrooke, QC J1K 2R1, Canada; Hu.Zhang@usherbrooke.ca (H.Z.); feidehai@163.com (S.G.); Shangyi.Fu@usherbrooke.ca (S.F.); 2 School of Material Science & Engineering, Beifang University of Nationalities, Yinchuan 750021, China

**Keywords:** near-infrared light-sensitive polymer, block copolymer hydrogel, thermosensitive polymer, upper critical solution temperature, photothermal effect

## Abstract

We report a near-infrared (NIR) light-responsive hydrogel that is capable of undergoing the gel to sol transition upon 785 nm light exposure based on a photothermal effect. The new hydrogel design relies on loading gold nanorods (AuNRs) in an ABA-type triblock copolymer, namely P(AAm–*co*–AN)–*b*–PDMA–*b*–P(AAm–*co*–AN), where P(AAm–*co*–AN) stands for a random copolymer of acrylamide and acrylonitrile that exhibits an upper critical solution temperature (UCST) in aqueous solution and PDMA is water-soluble polydimethylacrylamide. At solution temperature below UCST, the insoluble P(AAm–*co*–AN) blocks lead to formation of hydrogel of flower-like micelles. When the hydrogel is exposed to 785 nm NIR light, the absorption due to the longitudinal surface plasmon resonance of loaded AuNRs generates heat that raises the hydrogel temperature above UCST and, consequently, the gel-to-sol transition. The NIR light-triggered release of a protein loaded in the hydrogel was found to display a switchable fashion.

## 1. Introduction

Among stimuli-responsive polymer hydrogels, photosensitive bulk, micro- and nanogels have distinct features enabled by the use of light to stimulate desired changes [1,2,3,4,5,6,7,8,9,10,11,12,13]. Just like other photoresponsive material systems, the main advantages are remote activation and spatiotemporal control of the stimulation [14,15]. Basically, all photoresponsive hydrogels are based on either some sort of photochemical reaction [1,2,3,4,5,6] or photothermal effect [7,8,9,10,11,12,13], and light-induced hydrogel changes generally are volume transition (shrinking or swelling) [5,8,9,10,11,12,13] or gel-sol transition (loss of network structure and jellification) [1,2,3,4,6,16]. An important area of applications for hydrogels is in the biomedical field. For photoresponsive hydrogels, the excitation wavelength has long been known as a challenging issue. Near-infrared (NIR) light (wavelengths roughly 700–1000 nm) is preferred because of its deeper tissue penetration and less detrimental effect on healthy cells as compared to ultraviolet (UV) light. Making NIR-responsive hydrogels, however, is not trivial, largely due to the lack of appropriate photochemical reactions (e.g., photocleavage) induced by one-photon absorption of NIR light [17,18,19]. In recent years, some solutions to this problem have been proposed. For example, we previously reported the loading of upconverting nanoparticles (UCNPs) in a UV-degradable hydrogel [20]. Upon NIR (980 nm) excitation, UCNPs emit UV light from the interior of the hydrogel that, in turn, are absorbed by the polymer, which activates a photocleavage reaction to cut the crosslinker segments and thus leads to the gel–sol transition. On the other hand, Kumacheva et al. showed that by loading gold nanorods (AuNRs) in a thermosensitive hydrogel having a lower critical solution temperature (LCST), NIR light could induce volume shrinkage of the hydrogel [8,9]. In this case, when the hydrogel, with the polymer at *T* < LCST, is exposed to NIR at a wavelength near the longitudinal surface plasmon resonance (SPR) of AuNRs, the light absorption generates heat released inside the hydrogel that can raise the temperature above LCST, thus resulting in the hydrogel volume shrinkage as the polymer becomes insoluble in water. More recently, some other NIR photothermal agents, such as graphene oxide [21], gold-silica nanoshell [22], carbon nanotube [23], semiconducting polymer [24], and polymer nanoparticles [25] were also employed to induce the de-swelling of the LCST-type hydrogels, resulting in the release of encapsulated drug. These examples present the two typical general strategies and are representative of photochemical reaction- or photothermal effect-based NIR-responsive hydrogels. However, to our knowledge, only a few studies were reported thus far on NIR light-sensitive hydrogels that can undergo the gel–sol transition under photothermal effect [26,27]. Basically, it involves the combination of a NIR photothermal sensitizer with either a supramolecular hydrogel bearing thermo-degradable crosslinker or a hydrogel displaying a positive thermosensitivity. The principle behind those studies is the breakup of the physical cross-links upon exposure of NIR, which requires a temperature increase above a critical temperature. In the case of the supramolular hydrogel, it needs to be heated above 50 °C to change from the solid state to the sol state [26]. In the case of the hybrid hydrogel of positive thermosensitivity, the phase transition temperature is around 45 °C [27]. Herein, we report a new kind of NIR-responsive hybrid hydrogel composed of a polymer displaying an upper critical solution temperature (UCST) and gold nanorods (AuNRs). The UCST can be adjusted depending on the application and, in the present study, to be slightly above the body temperature (37 °C) to demonstrate NIR light-triggered release. 

## 2. Materials and Methods

All reagents were purchased from Sigma-Aldrich (Oakville, ON, Canda). *N*-acrylamide (AAm) was recrystallized in chloroform prior to use; acrylonitrile (AN) and *N*,*N*′-dimethylacrylamide (DMA) were purified by passing through a column filled with basic alumina. 2,2′-Azobis(2-methylpropionitrile) (AIBN) was purified by recrystallizations from ethanol. The RAFT chain transfer agent, *S*,*S*′-bis(α,α′-dimethyl-α″-acetic acid)trithiocarbonate (BTC), was synthesized according to a reported method [28]. Other chemicals were used as received. Dimethyl sulfoxide (DMSO) was HPLC grade. Dialysis membrane tube with a molecular weight cut-off (MWCO) of 3000 was purchased from Spectrum Laboratories (Rancho Dominguez, CA, USA).

In order to synthesize the triblock copolymer with the UCST block at the two ends, P(AAm–*co*–AN) was first synthesized using RAFT polymerization, with a trithiocarbonate chain transfer agent (CTA). Then, the P(AAm–*co*–AN) was used as macro-CTA to polymerize dimethylacrylamide, resulting in the triblock copolymer P(AAm–*co*–AN)–*b*–PDMA–*b*–P(AAm–*co*–AN).

**Figure 1:**
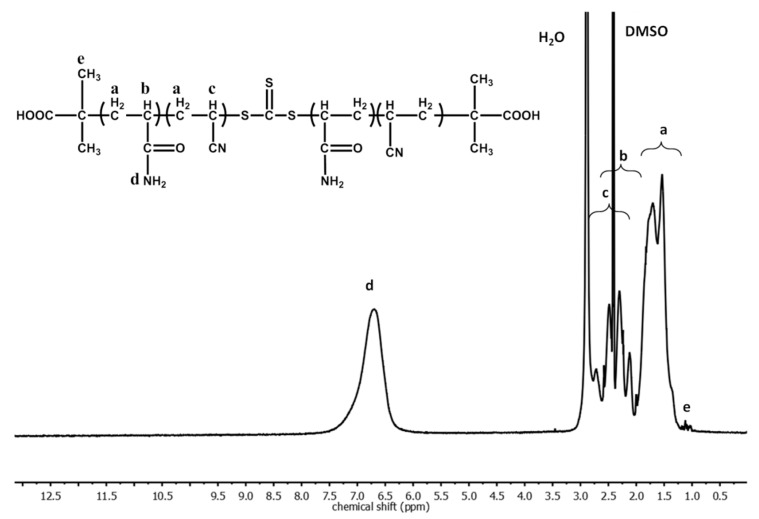
^1^H NMR Spectrum of the random copolymer P(AAm–*co*–AN) in *d*_6_-DMSO.

Synthesis of macro-CTA, P(AAm–*co*–AN). A 25 mL round-bottom flask was charged with AAm (3140 mg), AN (700 mg), BTC (16.2 mg), AIBN (1.88 mg), and DMSO (13.47 mL). The mixture was degassed by three freeze-thaw cycles and then placed into an oil bath preheated to 70 °C. The polymerization was allowed to proceed for 3 h with the mixture under stirring. Afterwards, the polymer was purified by precipitation from the DMSO solution into methanol twice. To completely remove DMSO, the polymer re-dissolved in DMSO was dialyzed against de-ionized water for three days and then recovered by lyophilization. Using an infrared spectroscopic method [29], the AN content in the obtained P(AAm–*co*–AN) sample is about 32 mol % based on the ^1^H NMR spectrum shown in Figure 1. From size exclusion chromatography (SEC), *M*_n_ = 54,573 g/mol; PDI = 2.3.

**Figure 2:**
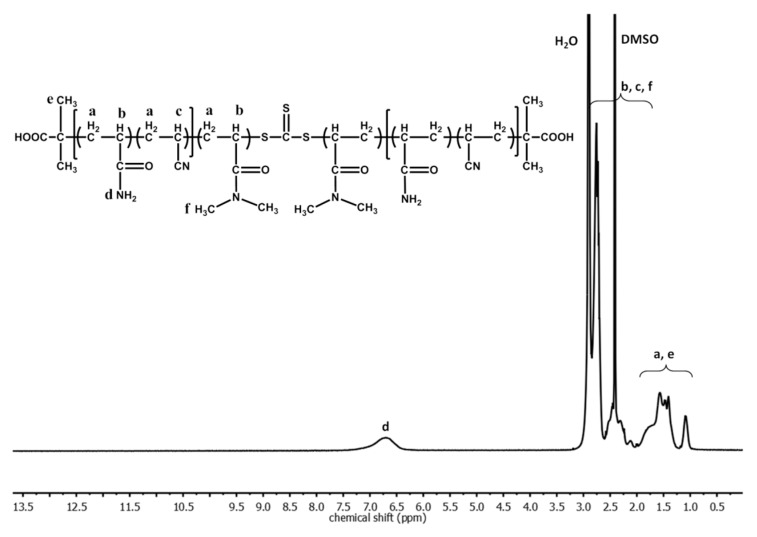
^1^H NMR Spectrum of the triblock copolymer P(AAm–*co*–AN)–*b*–PDMA–*b*–P(AAm–*co*–AN) in *d*_6_-DMSO.

Synthesis of P(AAm–*co*–AN)–*b*–PDMA–*b*–P(AAm–*co*–AN). DMA (1670.0 mg), P(AAm–*co*–AN) (500.0 mg), AIBN (1.1 mg), and DMSO (16.8 mL) were added into a 25 mL round-bottomed flask. The reaction mixture was degassed by pouring N_2_ flow for 1 h, then the flask was sealed and immersed in an oil bath thermostated at 60 °C for 6 h. It was then diluted with DMSO and directly dialyzed against deionized water for three days to remove unreacted DMA. The triblock copolymer was finally collected by lyophilization. From SEC, *M*_n_ = 108,586 g/mol; PDI = 2.4. Using the *M*_n_ and composition of the macro-CTA P(AAm–*co*–AN), the degrees of polymerization of all blocks could be estimated, yielding a triblock copolymer sample of P(AAm_261_–*co*–AN_165_)–*b*–PDMA_545_–*b*–P(AAm_261_–*co*–AN_165_). Figure 2 shows the ^1^H NMR spectrum of this triblock copolymer. 

Gold nanorods (AuNRs) were synthesized using a seed mediated growth procedure reported in the literature [30,31]. Briefly, gold seed solution was firstly prepared by dissolving 365 mg CTAB in 10 mL water in a round flask; HAuCl_4_ stock solution (25 mM, 100 μL) was then added to the CTAB solution and the mixture was vigorously stirred (1200 rpm) at room temperature. Afterwards, an ice-cold solution of NaBH_4_ (10 mM, 0.6 mL) was added quickly to the above mixture and the whole further stirred for 2 min. The color of the mixture changed immediately from yellow to brown after adding NaBH_4_, indicating the formation of gold seeds. These seeds were aged for 2 h before use. The second step is the growth procedure. CTAB (3644.5 mg) was dissolved in water (100 mL) in a 250 mL round-bottom flask. Other reagents were added to this solution in the following order: silver nitrate solution (10 mM, 0.7 mL), aqueous HAuCl_4_ stock solution (25 mM, 2.0 mL), HCl solution (1 M, 0.2 mL), and l-ascorbic acid (78.8 mM, 0.70 mL). This mixture was stirred gently (400 rpm) for 30 s. Afterwards, the seed solution (120 μL) was added, stirred for 30 s, and then left undisturbed overnight (15 h). The temperature of the growth step was kept constant at 29 °C all the time. The obtained AuNR solution (100 mL) was subjected to centrifugation, and the precipitated AuNRs were redispersed in water (5 mL) for further use.

^1^H NMR spectra were recorded on a Bruker 400 MHz spectrometer (Bruker Corporation, Billerica, MA, USA) using deuterated DMSO (*d*_6_-DMSO) as solvent at 100 °C. Size exclusion chromatography (SEC) measurements were carried out on Tosoh EcoSEC system (Tosoh USA, Inc., Grove City, OH, USA), equipped with three TSK-GEL Super AWM-H columns (6 mm × 150 mm), at 45 °C using DMSO containing 1.25 mg·mL^−1^ of LiBr as the eluent (flow rate: 0.3 mL/min) and poly (methyl methacrylate) (PMMA) as standards. Fluorescence emission spectra were recorded using a Varian Cary Eclipse fluorescence spectrophotometer (Agilent Technologies, Santa Clara, CA, USA). The UCST shift was investigated by monitoring the change in cloud point of a given polymer solution, which was taken as the inflection point of the transmittance (at 500 nm) vs. temperature curve. This curve was recorded on an Agilent Cary Series UV–Vis–NIR spectrophotometer (Agilent Technologies, Santa Clara, CA, USA). Each measurement was carried out by first cooling the solution from the used highest temperature at a constant cooling rate of 1.0 °C/min and then heating the solution back to the starting temperature at the same rate.

## 3. Results and Discussion

**Figure 3:**
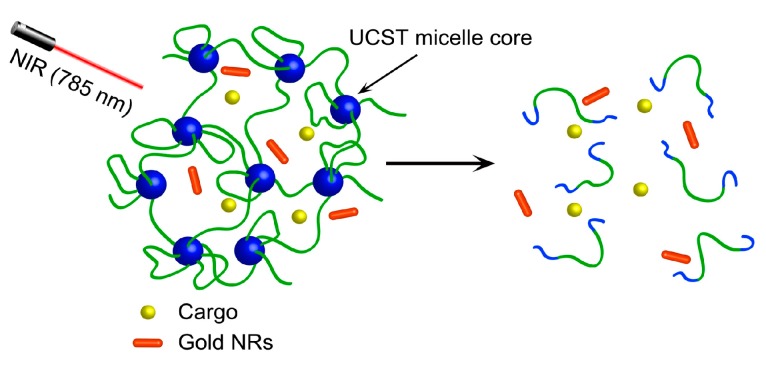
Schematic Illustration of NIR-light-triggered UCST gel–sol process due to heating by encapsulated AuNRs.

Figure 3 shows the hydrogel design. AuNRs are loaded in a hydrogel whose crosslinks are micelle cores constituted by a thermosensitive polymer displaying an upper critical solution temperature (UCST). The network structure is stable at *T* < UCST with the hydrophobic micelle cores, while upon NIR light exposure, heat released from AuNRs can raise the temperature above UCST and thus dissolve the micelle cores as the polymer becomes water-soluble. This gives rise to NIR induced gel-sol transition. Here, the positive thermosensitivity of UCST, opposite to LCST, is key because LCST polymers cannot lead to the gel-sol transition in this manner. To test the design, we synthesized an ABA-type triblock copolymer of P(AAm–*co*–AN)–*b*–PDMA–*b*–P(AAm–*co*–AN). As seen from its structure, the middle block is water soluble polydimethylacrylamide (PDMA) and the two end blocks are a random copolymer of acrylamide and acrylonitrile (P(AAm–*co*–AN)) whose UCST can easily be adjusted by varying the composition [29,32,33]. For the purpose of this study, we started by synthesizing P(AAm–*co*–AN) using RAFT polymerization, with a trithiocarbonate chain transfer agent (CTA). The P(AAm–*co*–AN) sample—was then used as macro-CTA to polymerize dimethylacrylamide, resulting in the triblock copolymer structure in inset of Figure 1.

**Figure 4:**
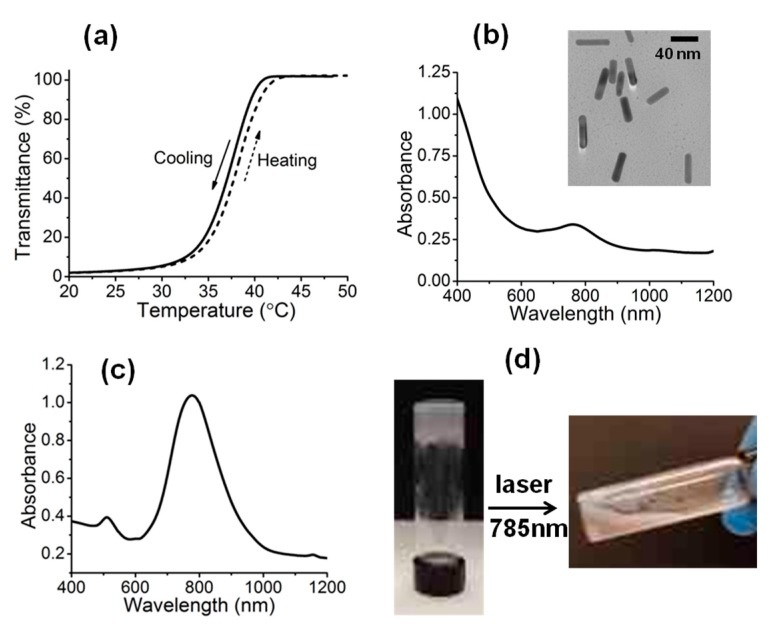
(**a**) Plots of solution transmittance (at 500 nm) vs. temperature of the triblock copolymer in water. (**b**) UV–Vis absorption spectrum of the UCST hydrogel encapsulated with AuNRs; inset: TEM image of the synthesized AuNRs. (**c**) Absorption spectrum of the synthesized gold nanorods (AuNRs) in water. (**d**) Gel–sol transition generated by applying laser.

The obtained sample, P(AAm_261_–*co*–AN_165_)–*b*–PDMA_545_–*b*–P(AAm_261_–*co*–AN_165_), is an amphiphilic triblock copolymer having an UCST of about 37 °C shown in Figure 4a, taken as the cloud point from the solution transmittance measurement. Formation of physical hydrogel of such ABA triblock copolymer is known [34,35,36,37,38,39]. Generally speaking, flower-type micelles can be formed in dilute solution with A-block core and B-block corona, but upon increase of polymer concentration hydrogel can be obtained. The gelation occurs as a result of ‘bridging’ chains that locate their end blocks in different micelles and lead to a network structure. In the present case, P(AAm–*co*–AN)–*b*–PDMA–*b*–P(AAm–*co*–AN) was first dissolved in water at 60 °C (200 mg/mL), together with cetyltrimethylammonium bromide (CTAB) stabilized AuNRs (1 wt % with respect to the polymer), synthesized according to a literature method [30,31]. Simply on cooling the solution to room temperature, AuNR-loaded hybrid hydrogel was formed. Figure 4b shows the UV–Vis absorption spectrum of the hydrogel. Although the weak transverse SPR peak near 520 nm is not visible due to the light scattering of the opaque hydrogel, the dominant longitudinal SPR peak around 780 nm is clear (both peaks are visible with AuNRs in water, Figure 4c). The absence of broadened longitudinal peak indicates a well-dispersed state of AuNRs inside the hydrogel. The inset in Figure 4b is a TEM image of the synthesized AuNRs, with an aspect ratio of 3.3. The NIR light-induced gel-sol transition is visually observable when the hydrogel is exposed to a continuous-wave laser at 785 nm (1 W), as shown by the photos in Figure 4d. The flowing sol state was obtained under the NIR irradiation, meaning that the photothermal effect could raise the hydrogel temperature above 40 °C. As the P(AAm–*co*–AN) micelle core becomes soluble in water, the crosslinks are lost. Obviously, the same gel–sol transition can be obtained by direct heating of the hydrogel to *T* > UCST.

**Figure 5:**
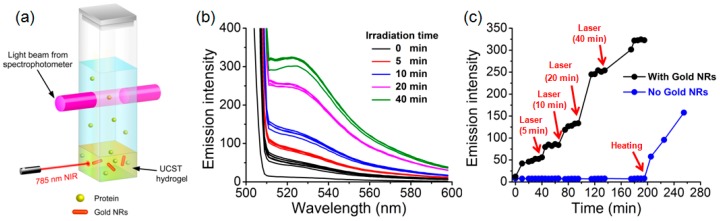
(**a**) Setup used to detect protein diffusing from the hydrogel into the aqueous solution after 785 nm laser irradiation. (**b**) Fluorescence emission spectra of the protein recorded after exposing the hydrogel to laser light (1 W) for a certain amount of time (indicated). (**c**) Plots of the fluorescence emission intensity vs. NIR irradiation time, showing a temporal control of the protein release by turning the NIR laser on and off.

Biomacromolecules can easily be loaded in in the hybrid hydrogel. Fluorescein isothiocyanate conjugated bovine serum albumin (FITC-BSA, 0.033 mg) was dissolved in the triblock copolymer/AuNR solution (1 mL) at 60 °C; the solution placed in a quartz cuvette was cooled to room temperature to form a piece of hydrogel at the bottom. As depicted in Figure 5a, after adding water (2 mL) into the cuvette, fluorescence emission spectrum of FITC-BSA could be recorded to monitor the release of the enzyme from the hydrogel exposed to the 785 nm laser from a side. Figure 5b shows the emission spectra (λ_ex_ = 488 nm) of the hydrogel after NIR irradiation for a certain amount of time. The apparent step-wise increase of the fluorescence intensity, indicating more protein molecules released from the hydrogel into water through diffusion, occurred following an NIR irradiation. Figure 5c plots the maximum emission at 523 nm as a function of time, with the NIR irradiation times marked. When fresh water was added to immerse the hydrogel, the first spectra recorded indicate the presence of FITC-BSA in the solution prior to NIR exposure, implying that some protein molecules were not entrapped by the hybrid hydrogel. The amount of protein remained basically unchanged until 5 min NIR irradiation was applied (no spectral taking during the irradiation). Then, following each NIR irradiation, a jump of the amount of released protein was detected, while no significant release continued after the NIR exposure. The results indicate an NIR light induced release of FITC-BSA in an on-off fashion. In other words, during NIR irradiation, the gel–sol transition occurs and the reduced viscosity allows more protein to diffuse into the solution. However, once NIR is turned off, the fast cooling results in the reverse sol-gel transition that prevents further release of the protein from the hydrogel. It should be noted that with the used setup (Figure 5a), no stirring was used to facilitate the release. A control test was carried out to confirm that the observed NIR-induced on-off release of the protein was originated from the AuNR-enabled photothermal effect. FITC-BSA was loaded in the hydrogel without AuNRs under otherwise the same conditions, and the loaded hydrogel was subjected to the same NIR irradiation sequence and times. As seen from the result also in Figure 5c, the entrapping of the protein appeared better than with the hybrid hydrogel (prior to NIR exposure) and the repeated NIR irradiations resulted in no release. The release occurred only when the hydrogel was heated to 50 °C, above UCST of the P(AAm–*co*–AN) micelle core for the gel–sol transition.

## 4. Conclusions

To summarize, we have prepared an ABA-type triblock copolymer hydrogel that, for the first time, can undergo NIR light-induced gel–sol transition due to the photothermal effect enabled by the SPR absorption of a small amount of loaded AuNRs. The key to the block copolymer design is that the two end blocks are a thermosensitive polymer exhibiting an UCST, so that the hydrogel is formed at *T* < UCST with the micelle cores acting as crosslinks but converts onto sol upon NIR irradiation that raises the temperature above UCST and thus dissolves the micelles. Moreover, we showed that simultaneous entrapping of AuNRs and FITC-BSA, a macromolecular enzyme, in the hydrogel could easily be obtained by cooling the solution of the triblock copolymer P(AAm–*co*–AN)–*b*–PDMA–*b*–P(AAm–*co*–AN) with AuNRs and FITC-BSA from *T* > UCST (60 °C) to *T* < UCST (room temperature). We found that the release of FITC-BSA occurs when the hydrogel is under NIR irradiation but halts once the NIR light is tuned off. This switchable release is due to a reversible gel–sol transition arising from heating above UCST and cooling below UCST when the NIR laser is switched between on and off states, respectively. Our study thus demonstrates a new application of UCST block copolymers that is undoable with LCST polymers. 

## Statement

Portions of this work were presented and published in Hu Zhang’s Ph.D. thesis entitled with “Étude de nouveaux polymères thermosensibles en solution aqueuse et de leur changement de solubilité induit par le pH”.
